# Implementation of PRRSV status classification system in swine breeding herds from a large integrated group in Spain

**DOI:** 10.1186/s40813-019-0134-9

**Published:** 2019-12-15

**Authors:** D. Torrents, J. Miranda, R. Pedrazuela, P. C. Gauger, A. Ramirez, D. C. L. Linhares

**Affiliations:** 1 Laboratorios Hipra S.A, Av de la Selva 135, 17170 Amer, Girona Spain; 2 Veterinary Diagnostic and Production Animal Medicine, Iowa State University College of Veterinary Medicine, 1800 Christensen Drive Ames, Iowa, 50011-1134 USA

**Keywords:** Genetic diversity, Sows, PCR, Stability, Mass vaccination

## Abstract

**Background:**

Porcine Reproductive and Respiratory Syndrome (PRRS) is an endemic swine disease causing significant productive and economic losses. Knowledge of PRRS epidemiology is crucial to develop control strategies against this disease. In that regard, classifying farms according to PRRS virus (PRRSV) shedding and exposure, and understanding key drivers of change in status over time, provides great applied knowledge for developing disease control programs. In most European countries, PRRSV monitoring is performed most frequently at the individual farm level although criteria selected for monitoring varies among different regions and farms. The aim of this study was to implement a systematic monitoring program for PRRSV in Spanish sow farms. Breeding herds were classified according to a standardized PRRSV infection status using sampling programs and terminology currently adopted in the United States (US), which allowed an evaluation of PRRSV epidemiology in a large integrated Spanish group during a one-year study period (February 2017–March 2018).

**Results:**

Fifteen farms achieved a stable PRRSV status after the first 4 consecutive samplings and 20 farms were classified as unstable. One of the farms maintained a stable status throughout the duration of the whole monitoring period.

Among the 20 farms classified as unstable at the beginning of the monitoring protocol, 9 farms (45%) never reached the stable status and 11 farms (55%) reached stable status afterwards during the monitoring study period.

From PRRSV PCR positive pools, there were 47 different PRRSV nucleotide sequences from 24 different farms. More than one PRRSV sequence was obtained from 15 farms. In the farms with more than one sequence detected, we observed recirculation of the same PRRSV field strain in 7 farms and introduction of a different PRRSV strain in 5 farms and both events in 3 farms.

**Conclusions:**

Systematic monitoring for PRRSV in breeding herds established a basis of knowledge of PRRSV epidemiology at the farm level and provided key data to classify farms according to PRRSV exposure and shedding status. These data allow further evaluation of the impact of the PRRSV farm status on production and economic performance in breeding herds and additional investigation of factors related to PRRSV epidemiology.

## Background

Porcine Reproductive and Respiratory Syndrome (PRRS) is a swine disease causing significant productive and economic losses in pig farms due to reproductive failure in breeding females and respiratory distress in pigs of different ages [1–3]. PRRS epidemiological knowledge at farm or regional levels and farm classification according the PRRS virus (PRRSV) status are key points necessary to develop appropriate control strategies for this disease [4, 5]. In 2010, the American Association of Swine Veterinarians (AASV) Board of Directors approved a herd classification system for describing the PRRSV status of herds based on determining both shedding and exposure status of the herd [4]. In this classification, four main PRRSV status categories were described: 1) Positive Unstable, 2) Positive Stable, 3) Provisional Negative, and 4) Negative, based on the detection of PRRSV RNA by RT-PCR, and anti-PRRSV antibodies by ELISA in serum samples following a standardized sampling protocol. Nowadays, this classification is commonly used in the United States as a guideline for PRRSV monitoring in breeding herds, and many farms involved in local or regional PRRS control programs apply it systematically.

In Europe, PRRSV is endemically present in almost all countries with the exception of Norway, Finland, Sweden, and Switzerland, which are considered PRRSV-free countries [6]. Despite PRRS diagnostic assays being fully available in most European countries, PRRSV monitoring is performed most frequently at the individual farm level and following different criteria among different regions and farms. Therefore, systematic and periodic monitoring of PRRSV in Europe is lacking, which limits the epidemiological knowledge on PRRSV at regional or national levels. Moreover, at the farm level, the lack of systematic monitoring of PRRSV leads to irregular and non-standardized information about the PRRSV status of farms, which makes it difficult to understand if there had been any progress towards the negative impacts of PRRS or improved control over time.

The aim of this study was to establish, for the first time, a systematic monitoring program for PRRSV in Spanish sow farms. Breeding herds were classified according to a standardized PRRS status using terminology currently adopted in the US swine industry, which allowed an evaluation of the evolution of PRRS epidemiology in a large integrated Spanish group during a one-year study period.

## Material and methods

### Study design

This was a prospective field study which enrolled 35 Spanish breeding herds between February to March 2017 for a 12 months PRRSV monitoring with samples collected each month during the study period. Before starting the monitoring study period, general information of each farm was collected: farm ID, geographic location, herd size, production system and gilt replacement system.

### Study population

All 35 breeding herds (76,800 sows) from one large integrated group located in Spain were enrolled in the study. At the time of the study this group was performing an expansion plan including increasing farm capacities and recent integration of new breeding farms into the system. Farm size ranged from 550 to 3900 sows, and all farms were considered positive to PRRSV, *Mycoplasma hyopneumonia*e, *Actinobacillus pleuropneumoniae* and Influenza A virus at the beginning of the study period. All farms were located in North-East Spain covering 3 autonomous regions: Navarra (3 farms), Aragon (25 farms) and Catalunya (7 farms) and four different swine genetics were used in the system. Additional individual farm information is summarized in Table 1.

**Table 1:** Demographic information of the farms included in the study

Farm	Location (Spanish region)	Sows	Genetic code	Production system^a^
1	Catalunya	3000	A	S1
2	Aragon	1200	A	FTF
3	Catalunya	550	A	FTF
4	Catalunya	3000	A	S1
5	Catalunya	1000	A	S1 + S2
6	Aragon	750	A	S1 + S2
7	Catalunya	3500	A	S1
8	Catalunya	1100	A	S1
9	Aragon	550	A	S1
10	Aragon	1080	A	S1
11	Aragon	800	A	S1
12	Aragon	550	A	S1
13	Aragon	2800	B	S1
14	Aragon	2580	A	S1
15	Aragon	3000	B	S1
16	Aragon	3000	B	S1
17	Aragon	3500	B	S1
18	Aragon	2300	C	S1 + S2
19	Aragon	2400	C	S1 + S2
20	Aragon	2800	B	S1
21	Aragon	1200	A	S1
22	Catalunya	3500	A	S1
23	Aragon	2400	C	S1
24	Aragon	3300	B	S1
25	Aragon	2600	B	S1
26	Navarra	2900	C	S1 + S2
27	Navarra	2900	C	S1 + S2
28	Aragon	3300	C	S1
29	Aragon	620	C	S1
30	Aragon	2800	D	S1 + S2
31	Aragon	950	C	S1 + S2
32	Aragon	3900	B	S1
33	Aragon	3500	B	S1
34	Navarra	2000	B	S1
35	Aragon	1500	B	S1

^a^S1: Breeding farm only; S1 + S2: Breeding and Nursery sites in the farm; FTF: Farrow-to-Finish farm

### Diagnostic monitoring protocol

A systematic PRRSV monitoring for the classification of PRRSV status was designed based on the AASV guidelines [4]. More specifically, study farms adopted a diagnostic monitoring protocol, which consisted of individual blood sampling of 30 due-to-wean piglets every 4–6 weeks. Piglets were selected according the following criteria: one piglet per litter, preferably low-weight/weak piglets, and preferably from first parity sows. Serum from individual samples were pooled (5 pools of 6 samples), and tested for PRRSV RNA by RT-PCR using previously validated assays [7]. In farms where piglets were vaccinated at sampling time, samplings were carried out on non-vaccinated piglets located in separate rooms from vaccinated piglets.

Differently from the sampling protocol proposed in AASV guidelines, we did not selected male piglets since castration was not carried out in any of the study farms. So no higher PRRSV prevalence should be expected in males due to the iatrogenic transmission related to castration procedure. At the same time, pooling of individual samples in our study (5 pools of 6) also slightly differed from the protocol proposed in AASV guidelines (6 pools of 5). This difference did not compromise significantly the sensitivity of the protocol since a minor change of 1 to 2 units of Ct values should be expected.

### PRRSV status classification

The study started under the assumption that all PRRSV positive farms were positive but unstable, since no previous systematic PRRS diagnostics were available. Farms were considered a positive stable (PS) status after 4 consecutive negative PCR tests for all tested pools. When at least one pool was PCR-positive, farms remained in the positive unstable (PU) status. Similarly, farms that reached PS status during the study period turned PU when at least one subsequent pool was PCR-positive. On the other hand, PU farms changed to PS when they achieved 4 consecutive PCR-negative samplings. In this case, time to PS status was established starting at the time of the first PCR negative sampling in the series. To describe changes in PRRSV status over time, status of the farm was established based on the result of the most recent PCR sampling.

### Genetic diversity of PRRSV

Selected PCR-positive results were submitted for PRRSV open reading frame 5 (ORF-5) nucleotide sequencing by the Sanger method [8], which allowed description of PRRSV genetic diversity among study herds including the proportion of vaccine-like and field-type PRRSV. PRRSV ORF-5 sequences were analyzed, and contingency table and phylogenetic tree were determined using Geneious 11.1.5 software (Biomatters LTD, NZ). Selection of positive pools for sequencing was based on the PCR cycle threshold (Ct) value in order to maximize the success rate. More specifically, PCR-positive pools were considered eligible for sequencing when the Ct value was below 32, according to the history of success of the veterinary diagnostic laboratory that performed the sequencing. From this eligibility criteria, we selected the earliest and the latest positive sampling for each farm with Ct value below 32 in order to identify the circulating PRRSV at the beginning and at the end of the study period, and to assess potential PRRSV genetic diversity between these two time points. Similarly, positive samples with Ct values below 32 were considered eligible for PRRSV ORF-5 sequencing when PS farms shifted to PU during the monitoring period and also, periodically (every 3–4 months) for farms that kept PU status for > 4 months. PRRS viruses were considered MLV-vaccine like when they had at least 97% similarity with any known PRRS MLV vaccine sequence. Likewise, PRRS viruses were considered ‘field-type’ when nucleotide similarity to PRRS MLVs were less than 97%.

### Epidemiological data collection

Complementary information related to PRRS vaccination practices in each farm was collected through an interview of farm staff at every sampling time. This interview included questions about vaccination events performed in the farm prior to first sampling at the beginning of the study and since the last sampling visit for subsequent sampling times, recording last vaccination date and type of vaccine for either sows or piglets. In the event piglets were vaccinated at the farm, sampling was performed from the oldest piglets present, but not yet vaccinated and allocated to lactation rooms where none of the piglets were vaccinated.

### Statistical analyses

Descriptive statistics were used to document the proportion of herds in each PRRSV status category, and changes of PRRSV status over time. Moreover, a PRRSV phylogenetic description was conducted to report the PRRSV genetic diversity within study herds.

## Results

**Fig. 1:**
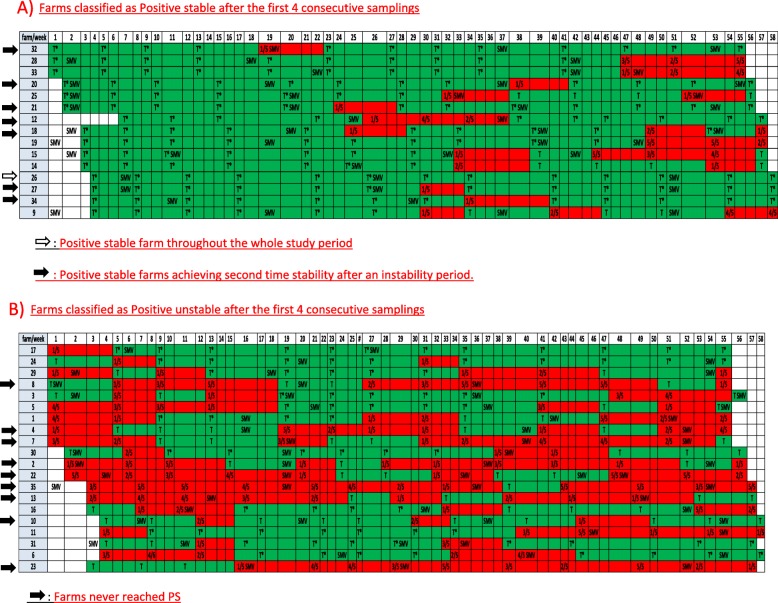
**a** Farms classified as Positive stable after the first 4 consecutive samplings Positive stable farm throughout the whole study period Positive stable farms achieving second time stability after an instability period. **b** Farms classified as Positive unstable after the first 4 consecutive samplings Farms never reached PS. **a** & **b** Summary of PRRSV RT-PCR results, and breeding herd PRRS status classification between February 2017 and March 2018. Farms are displayed in rows and study weeks in columns. Cell codes: x/5: PCR positive pools/5 tested pools; T: negative PCR test and positive unstable status; T*: negative PCR test and positive stable status; SMV: Sows mass vaccination. Red cells: most recent testing PCR-positive; Green cells: most recent testing PCR-negative

A total of 13 samplings per farm were performed during the one-year monitoring period in all 35 farms in the study. Results of each sampling and farm classification according the PRRSV status are displayed in Fig. 1. Fifteen farms (42.8%) reached PS status after the first 4 consecutive samplings and 20 farms (57.1%) were classified as PU. Just one of the PS farms at the beginning remained classified as PS during the entire monitoring period (Farm 26) and 14 farms shifted to PU. Additionally, 7 of these 14 farms reached PS status a second time (Fig. 1a). Among the 20 farms classified as PU at the beginning, 9 farms (45%) never reached PS status and 11 farms (55%) reached PS status at one point, but just 1 of these maintained the PS status for the rest of the monitoring period. The other 10 farms failed to maintain PS status and returned to PU status again (Fig. 1b).

**Fig. 2:**
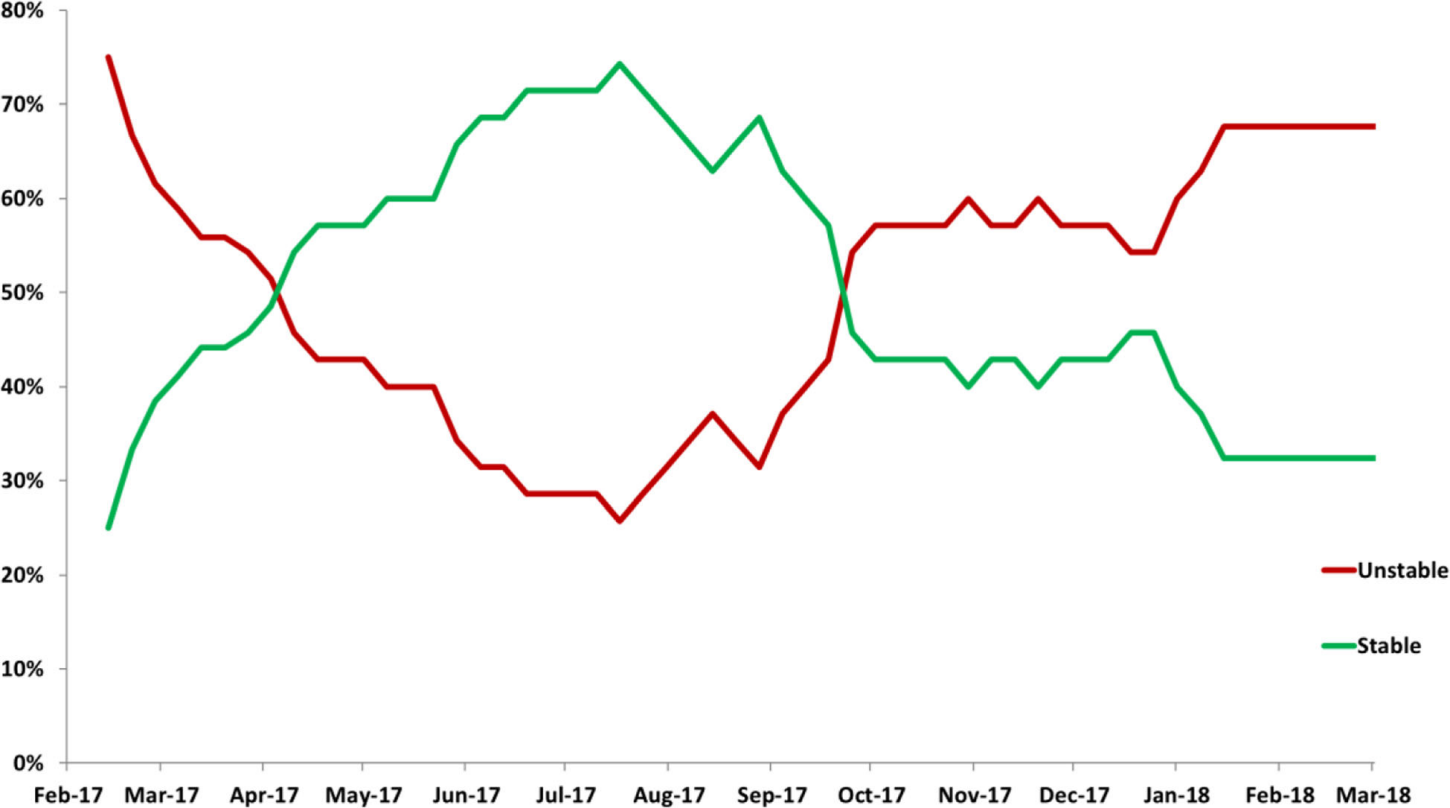
Evolution of the percentage of Unstable (PU) and Stable (PS) farms during the PRRSV monitoring period (February 2017 to March 2018)

Taken together, just 10 farms of 35 (28.6%; 1 PS and 9 PU) kept the same status for the entire study period, and 25 farms (71.4%) changed their PRRSV status one or more times. Throughout the study period, the percentage of PS farms (Fig. 2) increased from the beginning of the study (45.7%) until mid-July of 2017, where it reached the maximum (74.3%). Afterwards, this percentage dropped until January 2018 where it reached its minimum (32.4%).

According to the collected data related to vaccination practices in each farm along the study period, in all farms, sow mass vaccinations (SMV) with a MLV PRRS vaccine (UNISTRAIN®PRRS, Hipra, Spain) was administered. In most of farms (*n* = 20 farms) a 3-times-a-year SMV program was followed, scheduled around February, June, and October. However, in some farms an irregular program (*n* = 13 farms) was implemented or additional SMV in the event of PRRS clinical outbreak (n = 2 farms) was applied. Additionally, vaccination of piglets at 2–3 weeks of age with the MLV PRRS vaccine was carried out throughout all the study period in farms 1 and 4; and occasionally in 6 other farms (farms 7, 15, 16, 17, 22 and 31).

**Fig. 3:**
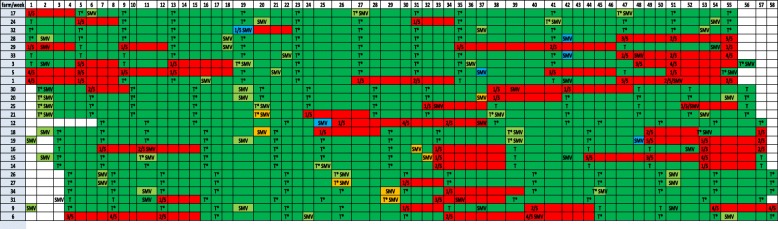
Summary of PRRSV RT-PCR results in farms where sows’ mass vaccination (SMV) was applied at PRRS stable status time (PS) (*n* = 58). Farms are displayed in rows and study weeks in columns. Cell codes: x/5: PCR positive pools/5 tested pools; T: negative PCR test and positive unstable status; T*: negative PCR test and positive stable status; SMV: Sows mass vaccination. Cell colors: Red cells: most recent testing PCR-positive; Dark green cells: most recent testing PCR-negative; Light green cells (SMV): SMV applied at PS without subsequent positive PCR test (*n* = 44): Blue cells (SMV): SMV applied at PS with subsequent positive PCR test related to field virus infection (*n* = 6); Orange cells (SMV): SMV applied at PS with subsequent positive PCR test with no ORF-5 nucleotide sequence available (*n* = 8)

Taking into account all 35 farms, during the study period we registered 126 SMV events, from which 58 (46%) were carried out during PS status time (Fig. 3). After SMV under PS classification, there was no detection of PRRSV by RT-PCR in weaning piglets on the next sampling in 44 out of 58 SMV (75.9%) events. For the other 14 SMV events (24.1%), PCR positive results were obtained in the next sampling after SMV. However, in 6 of these 14 cases (42.9%), the positive PRRSV PCR was related to the presence of PRRSV field strains, indicating a possible new recirculation or new introduction of PRRSV in the farm. For the other 8 SMV events where we obtained a PRRSV PCR positive result in the next sampling, ORF-5 sequence of PRRSV was not possible due to high Ct values, but in all cases positive PRRSV PCR results were limited to the immediate next sampling and not observed in subsequent samplings.

**Fig. 4:**
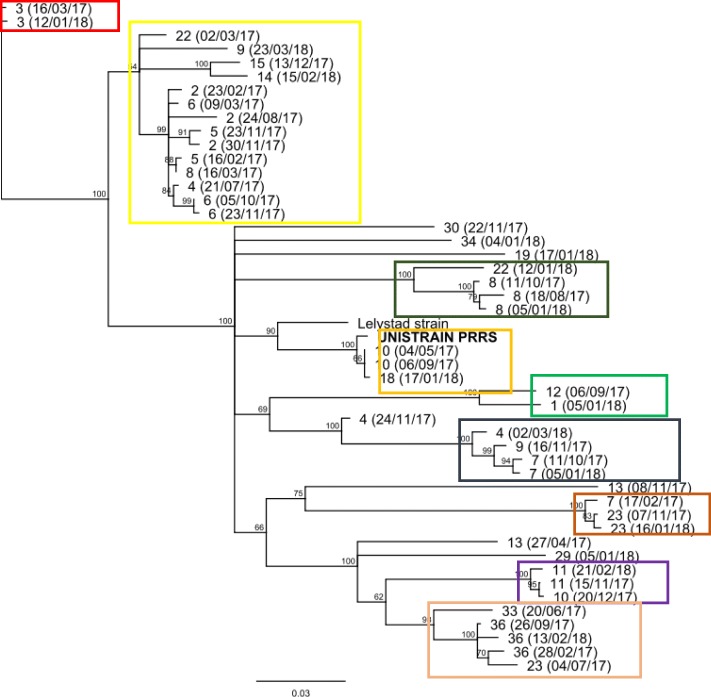
Phylogenetic tree based on complete PRRSV ORF5 gene nucleotide sequence of 47 PRRSV field strains and the the modified live virus (Unistrain®PRRS) used for vaccination in the study herds. The tree was generated using Neighbor-Joining method with Geneious Tree builder with bootstrats (× 1000 replicates) > 50%. The scale bare indicates the genetic distance. Different clusters are marked with different colored boxes

From 51 eligible PCR positive pools, we obtained 47 different PRRSV nucleotide sequences from 24 different farms. Epidemiologically and according to sequence homology and phylogenetic analysis, we could define 9 different PRRSV clusters encompassing 40 of the sequences, but 7 sequences did not cluster with a particular group (Fig. 4). More than one PRRSV ORF-5 sequence was obtained in 15 farms, and 9 farms had just one PRRSV sequence identified. In the 15 farms with multiple sequences, we observed recirculation of the same PRRSV field strain in 7 farms; introduction of a different PRRSV strain in 5 farms and in 3 farms we observed both recirculation of the same PRRSV strain and introduction of a different PRRSV strain. Nucleotide sequences matching with MLV applied in the farms were found only in 2 farms (farms 10 and 18).

## Discussion

The present study provides, for the first time, complete results of a 1-year systematic monitoring program of PRRS in breeding herds of a large integrated European swine production system. Systematic sampling of due-to-wean piglets every 4–6 weeks allowed us to establish the PRRSV status for each farm along the entire study period. Following PRRSV farm classification criteria proposed by AASV [4] just one farm classified as stable at the beginning of the study period remained stable for the entire 1-year study period (farm 26). Moreover, just one of the farms (farm 230) that reached PS status later during the study period kept this status until the end. On the other hand, 9 farms never reached PS status despite presenting different patterns of alternating PCR positive and negative results. This finding is in agreement with previous studies [9] demonstrating intermittent pattern of PRRSV detection by RT-PCR in herds undergoing PRRSV control and reinforcing the necessity of systematic and multiple-time sampling to establish a reliable PRRSV status of the farm. In PU farms, this intermittent pattern could be related to fluctuating PRRSV circulation levels over time getting closer to the 10% prevalence limit of detection of the sampling design [4]. In PS farms, occasionally PCR positive results were observed in the following sampling just after a PRRS SMV administration with MLV, indicating low levels of PRRSV vaccine strain circulation in due-to-wean piglets, which were not detected in the subsequent samplings. Unfortunately, due to the low PRRSV RNA content in these positive samples, identification of the PRRSV strain by nucleotide sequencing was not possible. Nevertheless, this detection made some PS farms shift to PU transitorily for several weeks. Detecting a PRRS MLV vaccine strain could mask the PS status of the farms, and thus affect the number of breeding farms which really remained PS for the whole study period.

Despite PRRS MLV showed the possibility of shedding and transmission between vaccinated and non-vaccinated animals under experimental conditions [10], in our study piglets’ PRRS vaccination at 2–3 weeks of age did not affect the PRRSV classification in any of the 6 farms when samplings were performed on piglets just before vaccination and located in separated barns from vaccinated piglets. This observation could indicate a very low rate of shedding and transmission of PRRSV MLV between piglets in lactation barns when vaccinated and non-vaccinated piglets are located in different rooms of the same barn, and vaccinated piglets remain in lactation areas no longer than 1 week after vaccination.

The increasing number of PS farms from the beginning of the study (February 2017) until July 2017 with a subsequent decrease from October 2017 until January 2018 could be related to the seasonal effects associated with winter that can increase the incidence of PRRS [11]. On the other hand, the decreasing number of PS farms also observed from July to October 2017 could be due to other epidemiologic factors [12] which should be further investigated; such as breeding herd replacement flow between farms [13].

Overall our findings suggest a highly dynamic PRRSV epidemiology within the farms with frequent new infections and recirculation of PRRSV. Most of the PRRSV strains identified through PRRSV ORF-5 nucleotide sequence were included in one of the clusters described in this study and presented a very close relationship with others strains found in different farms at different times, indicating a significant PRRSV transmission between farms; which are all owned by one company. This highly dynamic PRRSV epidemiology in this integrated group could be related to the geographical location of the farms in north-east Spain regions (Catalunya, Aragon and Navarra) with the highest density of pig farms in Spain and an area with the most intensive commercial pig and slaughtering activity in the country [14]. Moreover, an expansion of productivity was implemented in the company during the study period including increased farm capacities, high breeding herd replacement rates, and integration of new breeding farms into the system collectively made it difficult to establish and implement a global PRRS control strategy within the production system. Thus, the PRRS control strategies were tailored for each breeding herd.

## Conclusion

From February 2017 to March 2018, we observed a highly dynamic PRRSV epidemiology within different breeding herds from the same integrated group. Percentage of PS farms ranged from 32.4 to 74.3% during the study period. Over the 35 studied farms, 9 farms never reached PS status and just 1 farm was classified as PS for the whole study period. On the other hand, 25 farms shifted its PRRSV status at least once during the one-year period. Under PS status, SMV with the MLV PRRS vaccine showed minor interference with the PRRSV status classification. In just 8 out of 58 SMV events applied under PS status, we observed occasional positive PCR results in the following sampling just after a PRRS SMV. From positive-PCR samples, 47 different PRRSV strains were identified by ORF-5 nucleotide sequencing from 24 different farms. In 15 farms, more than one PRRSV strain was observed during the study period. Nine different PRRSV clusters encompassing 40 of the sequences were defined according to sequence homology and phylogenetic analysis. Overall, establishing this systematic monitoring program for PRRS set the basis for the knowledge of the PRRSV epidemiology at both the system group and farm level and provided key data for PRRSV status farm classification. These data will allow us to further evaluate the possible impact of PRRSV farm status on productive and economic performance of breeding herds, to further investigate factors related to PRRSV epidemiology and to plan strategic actions for the control of PRRS.

## Data Availability

The datasets used and/or analyzed during the current study are available from the corresponding author on reasonable request.
